# Powers and Immunities of Trade Unions

**Published:** 1913-07-12

**Authors:** 


### THE NATIONAL MEDICAL GUILD.

### Powers and Immunities of Trade Unions.

### Restraint of Trade.

Trade unions may operate in restraint of trade, other organisations may not. The exact meaning of restraint of trade is, however, a cause of stumbling to some people. Every person has a right in law to dispose of his labour at what wage he chooses in what market he chooses. In the same way he has an elementary right to drive on the wrong side of the road. But in both cases it has been found necessary for the good of the community to curb the individual liberty of the units which go to make the community. In the latter case it is effected by giving the courts certain powers over those who cause obstructions—which is the result of driving on the wrong side of the road. In the former case the effectual restriction is contained in the Trade Union Acts.

This restriction, by which a man may be hindered in his free disposal of his labour or capital, is one which removes the remedy which he would otherwise have against those who hindered him. The matter is best set out in an example.

Suppose that Dr. Smith goes to Welshtown as medical officer of a medical-aid institution which is objectionable, on account of the terms offered, to the local profession. He has an abstract right to do so. But the profession has also a right to hold out for what it considers fair terms—any profession has a moral right to do all in its power to ensure that its members have a proper return for their labour. Here are two rights in conflict—the right of Dr. Smith to do as he will with his labour, and the right of the profession to uphold a certain standard of remuneration.

In such cases it is a natural law that the good of the individual must bow to the good of the community, and the right of Dr. Smith is accordingly the one to suffer. The law, therefore, provides in the Trade Union Acts that Dr. Smith shall have no remedy against the local profession if they combine to prevent him earning a livelihood in his post. But the immunity is not quite so wide as would appear from the last statement, for the State, in putting a body of men above the law, requires guarantees that this immunity shall not be made to cover other Acts than those specified in the rules of the body which calls itself a trade union and avails itself of the advantages of registration as such. Thus the union must not slander Dr. Smith or say that about him which is not true, although it may seek to dissuade people from employing him. Moreover it cannot follow Dr. Smith into some other part of the country and there seek to evict him from similar employment unless the members of the profession in that locality are also members of a trade union, and desirous of attacking medical aid associations.

### Trade Disputes.

A trade dispute in relation to medicine is very difficult to define. There is no precedent on which to base a definition. In ordinary industrial disputes many persons are involved, and the whole of their means of livelihood is in jeopardy. They are, moreover, disputes between those who stand obviously in the relation of employers and employed. But in the case of Dr. Smith above the local profession were the employees of the resident population in a rather different sense, inasmuch as no one of them could say that any of his patients was under any definite contract to employ him. It is necessary in order to claim immunities that there shall be a trade dispute of some substance in progress. It is therefore obvious that this question of a definition of a medical trade dispute is one of some interest to the profession. It may, however, be stated that the decision in other countries has been in favour of the profession—*i.e.* they have not been interfered with, and the general tendency of the law is in the direction of affording larger powers of self-defence to professional organisations rather than of restricting them.

### Breach of Contract.

It is an offence to procure a breach of contract, and it is still more an offence to band together with others to procure wholesale breach of contract. Yet it is obvious that breach of contract may be the only effective reply to abuse of the terms of the contract by one of the parties thereto. Again an example will perhaps help matters. First we may take the example of Dr. Smith already dealt with. Suppose that the local practitioners were successful in persuading Dr. Smith (who, we will suppose, took the post in ignorance of the true position of affairs) to break his contract with the medical aid association which employed him. The association would have a grievance against these medical men and the courts would recognise this and award them suitable damages. But if the breach of contract was procured as part of the operation of a trade dispute, it is specifically stated no action shall lie.

The words of the Trade Disputes Act, 1906, 3, are as follows: "An Act done by a person in contemplation or furtherance of a trade dispute shall not be actionable on the ground only that it induces some other person to break a contract of employment, or that it is an interference with the trade, business, or employment of some other person or with the right of some other person to dispose of his capital or his labour as he wills." It is to be noted that some other cause of action might be pleaded and might be successful, although the defendant was a trade union official. Such other cause might be intimidation, but intimidation must be such that the intimidated has reason to fear actual bodily harm or violence to himself or family. To shake a big stick at a man is not intimidation unless he has reason to suppose that the stick will, as a matter of fact, be used, failing his compliance with requests. The medical profession is hardly likely to threaten or use violence of this sort, and, in consequence, is likely to be immune from actions based on alleged intimidation.

Watching and besetting is also a cause of action, but this again must be something essential. It is unlawful to follow a man from place to place in an unruly manner, or in such a way as to interfere with his personal liberty, but it is not unlawful to observe whether or no a medical man enters the premises of an unregistered dentist with a case of anæsthetic instruments in his hands. Anyone who gave evidence to that effect in support of a charge of "covering" would not render himself liable to a charge of watching and besetting. Here, again, the medical profession need not fear actions founded on alleged illegal actions committed in the course of an attempt to remove a man from a certain locality in which his presence was contrary to the welfare of the resident profession.

### Conspiracy.

Conspiracy is a banding together of persons to do a thing which need not of necessity be illegal if done by one alone. Thus one man may advise a friend as he will or an enemy. But if twelve people advise a man together, and in combination, it comes very near bringing undue pressure to bear. The advised may then plead that there is a conspiracy to cause him to follow the advice, perhaps against his better judgment.

On the other hand, the act may be unlawful even for one person, but, nevertheless, other elements are introduced by a combination for the same purpose. We have recently had an example of this in this country. Many persons saw fit to break the windows of shops and to commit arson and other crimes. Entirely individual action the organisation of the law is able to cope with. But here was a case in which the persons who committed offences were often of no substance, so that no damages could be obtained from them. It was at the same time notorious that they were directed by a body which did possess funds and which incited them to crime. Hence this body was charged, not with committing crimes, but with conspiring to procure the commission of crimes. The element of conspiracy was introduced.

Let us take a medical example. Suppose that when Dr. Smith took the post at Welshtown the staff of the local hospital desired to render what assistance they might to the local practitioners. Let us suppose that they refused to attend such of Dr. Smith's patients as might come to the hospital or to meet Dr. Smith in consultation. Each individual of that staff had a right to refuse any patient he chose, and to refuse to meet in consultation any doctor he chose. But in this case it is obvious that they banded together to do certain things, and that by virtue of all of them agreeing to the same course of conduct, Dr. Smith was seriously hindered in his earning of a livelihood. Here are all the elements of a conspiracy. No one of them is doing an illegal act, but the whole of them doing the same act made the complexion of matters, and especially the result of their acts, very different. Dr. Smith, therefore, had a good cause of action against them on the grounds stated. But let us suppose that they were all members of a trade union, and that a trade dispute was in progress, then no action against them on these grounds would be entertained by any court. This is a very important immunity of trade unions.

### Maintenance of Members of Public Bodies.

It will be remembered that prior to the Osborne judgment trade unions were in the habit of supporting members of Parliament, who were then unpaid as far as the State was concerned, out of trade-union funds. Nor did they distinguish the source of these funds.

It thus happened that men were obliged to subscribe to objects of which they did not approve as a condition of enjoying the other benefits of the unions.. This was not in accordance with the law of the land, and in the Osborne judgment this was made plain.

Trade unions were then no longer able to support members of Parliament except by voluntary contributions, and a very large number of their members were too lazy to care about contributing to objects of which they understood very little, and which did not result in immediate or tangible gain. The trade unions, therefore, brought pressure to bear on the Government, and the results were two. In the first place payment of members of Parliament by the State was inaugurated, and secondly a new Trade Union Act was passed. This was the Act of 1913.

It is often supposed that this Act enabled trade unions to revert to the *status quo ante* the Osborne judgment, but this is not the case. That position of affairs was, and still is, undesirable. It is by virtue of the law on this point that our own British Medical Association is unable to support a member of Parliament. It is not one of the objects for which the members of the British Medical Association subscribed, and it would be unfair to say to them that as a condition of membership they must allow their funds to be used in a way of which they do not approve.

But the 1913 Act does allow trade unions to support members of Parliament by contributions made for that purpose, whether that purpose was originally specified in their rules or not. These contributions must be collected from members in a definite manner. Thus each member must be asked whether he approves of political activities and given a chance of demanding exemption from contribution if he does not. A majority must declare in favour of political activity, or the union cannot undertake it at all. This gives ample safeguards, and under these conditions a union may support a member of Parliament.

There can be no doubt that the medical profession ought to have at least two members of Parliament under an obligation, in return for support, to ask questions and promote legislation for the benefit of the profession. There should be one on each side of the House so that we may be represented on the councils of both parties, not in the lobby as heretofore, but in committees and in places where they meet to do business.

